# Transactions of American Dental Association

**Published:** 1863-06

**Authors:** 


### TRANSACTIONS OF AMERICAN DENTAL ASSOCIATION.

The transactions of the last meeting of that body are now ready for distribution to all members of the Association, who are not in arrears for annual dues; and to all members of local societies, who are represented, or desire to be, in the National Association, according to provisions made for that purpose. The resolution of the Association in reference to this matter is as follows, viz.: "The Committee of Publication shall duly notify the various ocal dental societies through the periodicals or otherwise in advance of such publication that their members are respectively entitled to receive each a copy of the proceedings thus published, by forwarding to the Committee the sum of two dollars."

Copies may be procured by addressing J. TAFT, Cincinnati, Ohio, or J. F. JOHNSON, Indianapolis, Ind. By order of the Committee.

### CORRECTION.

In the April No. of the Register, we stated that by a resolution the American Dental Association requested all local societies to report the numbers and names of members, &c., to the Secretary of the Association. This we did from memory. We find by reference to the proceedings that such reports were to be made to the "Publication Committee." These reports may be sent to any member of that committee. It consists of Drs. J. F. Johnson, Indianapolis, Ind.; J. Taft, Cincinnati, O.; Wm. A. Pease, Dayton, Ohio; B. Strickland, Cleveland, Ohio. It is desirable that all such reports be sent immediately. T.

### TEETH.

We have recently received a lot of teeth from the establishment of Cunn & Armstrong, for rubber work. These teeth are beautiful indeed, having many points of excellence; the form is good, the color right and the blending of shades unexceptionable. The manner in which the margin of the gum is formed round the necks of the teeth gives them a very natural appearance; both look just as though they had grown out of the gum in the natural way. We know if our market was well supplied with these teeth, large quantities would be sold. T.

### ARTICULATING WAX.

We have just received from S. S. White wax and paraffine, prepared in proper form for taking articulations. It is certainly very convenient. We have tried both, and are rather inclined to give the wax the preference. In taking an articulation, the time saved by using this is worth more than the price of a whole box of twenty five pieces. Everything should be used that would save time and diminish and facilitate labor. T.

